# Do common mental disorders decline over time in TB/HIV co-infected and HIV patients without TB who are on antiretroviral treatment?

**DOI:** 10.1186/1471-244X-13-174

**Published:** 2013-06-27

**Authors:** Amare Deribew, Kebede Deribe, Ayalu A Reda, Markos Tesfaye, Yohannes Hailmichael, Todd Maja

**Affiliations:** 1 Department of Epidemiology, Jimma University, Jimma, Ethiopia; 2 Brighton and Sussex Medical School, Falmer, Brighton, UK; 3 College of Public Health, Haromaya University, Harar, Ethiopia; 4 Department of Psychiatry, Jimma University, Jimma, Ethiopia; 5 Department of Health Service Management, Jimma University, Jimma, Ethiopia; 6 Department of Health Studies, UNISA, PO Box 392, Pretoria, South Africa

**Keywords:** TB/HIV Co-infection, Quality of Life, Common Mental Disorders, Ethiopia

## Abstract

**Background:**

The relationship between TB/HIV co-infection and common mental disorders (CMD) is not well investigated. A follow up study was conducted to assess the change in CMD over a 6-months period and its predictors among TB/HIV co-infected and HIV patients without TB in Ethiopia.

**Methods:**

A longitudinal study was conducted in 2009. A total of 465 HIV/AIDS patients without TB and 124 TB/HIV co-infected patients from four antiretroviral treatment (ART) centers in Ethiopia were recruited to assess CMD and quality of life (QoL). CMD and QoL were assessed at baseline and at six month using the Kessler-10 scale and the short Amharic version of the World Health Organization Quality of Life Instrument for HIV clients (WHOQOL HIV-Bref) respectively. Multivariate analysis was conducted using generalized estimating equations (GEE) using STATA to assess change in CMD and its predictors.

**Results:**

At the 6 month, 540 (97 TB/HIV co-infected and 455 HIV/AIDS patients without TB) patients completed the follow up and 8.6% (21% among TB/HIV co-infected and 2.2% among HIV patients without TB) lost to follow-up.

At baseline, 54.4% of TB/HIV co-infected patients had mild to severe mental disorder compared to 41.2% among HIV patients without TB. At the six month follow up, 18.1% of TB/HIV co-infected patients had mild to severe mental disorder compared to 21.8% among HIV patients without TB. The decline of the prevalence of any form of metal disorder was 36.3% among TB/HIV co-infected patients compared to 19.4% among HIV patients without TB (P<0.001).

QoL was strongly associated with CMD in TB/HIV co-infected patients and HIV patients without TB (β = −0.04, P<0.001) after controlling the effect of several confounding variables such as sex, income, WHO disease stage, duration on ART, CD4 lymphocyte count, adherence to ART and social support.

**Conclusion:**

The prevalence of CMD has significantly reduced particularly among TB/HIV co-infected patients over a 6 months period. Poor QoL is the major independent predictors of CMD. We recommend integration of mental health services in TB/HIV programs. Training of health care providers at TB/HIV clinics could help to screen and treat CMD among TB/HIV co-infected patients.

## Background

Neuropsychiatric conditions represent 13% of the total disease burden and it is the third largest cause of disability-adjusted life years lost (DALYs) after infectious and parasitic diseases (41%) and cardiovascular diseases (26%) [1].

The importance of mental health is estimated to increase in the next two decades. Mental health conditions such as depression are projected to become the leading burden of DALYs by the year 2030 [2,3]. Three-quarters of this burden occurs in the developing world where Tuberculosis and HIV are prevalent [2,3].

Tuberculosis (TB) and HIV co-infection comprises an enormous burden on individuals and health care systems particularly in resource constrained countries [4,5]. Recently, co-morbidities between mental health and infectious diseases and integration of these diseases in the primary health care are getting much attention. Few studies have documented the interaction between HIV/AIDS and mental health problems. The presence of mental disorder, particularly depression, predisposes individuals to unprotected sexual behavioral and drug abuse which are the major risk factors for HIV/AIDS infection [6]. On the other hand, HIV/AIDS infection causes psychological trauma and affect the nervous system directly resulting in depression, mania and other cognitive disorders [7-11]. Opportunistic infections as a result of HIV/AIDS [6] and anti-retroviral drugs particularly efavirenz can also cause mental health disorders [6].

Few studies have documented high prevalence of tuberculosis among individuals with mental health problems [12,13]. In addition, high prevalence of depression was recorded among tuberculosis patients compared to health controls [14,15].

The complex interaction among TB, HIV and mental disorders could affect the quality of life (QoL) of individuals [4,16,17]. Since the advent of ART, the incidence of HIV-associated dementia has halved [18] and the prevalence of opportunistic infection of the central nervous system decreased [18,19]. However, there are reports indicating rise in the incidence of HIV encephalopathy [19].

There is a dearth of literature on the mental health status of TB/HIV co-infected patients over time. We conducted a follow up study in Ethiopia to document the changes in prevalence of CMD and its predictors among TB/HIV-co-infected and HIV patients without TB over six months period.

## Methods

### Study settings and population

This is a longitudinal study with 6 month follow-up period. The baseline survey was conducted from February to April, 2009 to assess the prevalence of CMD among 465 HIV/AIDS patients without TB and 124 TB/HIV co-infected patients from the ART clinics of Adama, Nekemet and Jimma specialized hospitals in Ethiopia. The methodology and the baseline findings are published in a previous article [16]. In general, 465 patients without TB and 124 TB/HIV co-infected patients who were taking ART were recruited in the three hospitals to compare CMD among the two groups. The sample size was determined based on the following assumptions: 5% lower mean score of physical QoL life among TB/HIV co-infected patients compared to HIV patients without TB, 95%CI, a 1:3 ratio of HIV/TB co-infected patients versus HIV patients, and a 10% for non-response rate. All the TB/HIV co-infected patients were in the intensive phase of anti-TB treatment during the recruitment period. For each TB/HIV co-infected patients, 3 HIV patients without active TB were selected in the TB/HIV clinics using a simple random sampling technique. The exclusion criteria for both groups were age less than 15 years, the presence of an opportunistic infection or a known chronic illness like diabetes mellitus and hypertension.

### Data collection procedures and follow up

Diagnoses of TB and HIV at baseline were based on the national guideline [20]. Smear microscopy was the major diagnostic tool for pulmonary TB. TB lymphadenitis was diagnosed based on clinical parameters and cytological examination obtained by fine needle aspiration. TB was excluded from the HIV patients by a thorough symptom screening.

After recruitment, patients were appointed monthly to the ART clinics of the respective hospitals to take their medication (anti-retroviral and ant-TB drugs). During each visit, patients were thoroughly assessed by trained nurses for drug side effects, general health status and presence of symptoms of opportunistic infection including TB. CD4 lymphocyte count and WHO clinical staging were extracted from the patients’ record at baseline and at the 6^th^ month. CMD and QoL were measured at baseline and at the 6^th^ month of follow up.

Common Mental Disorder was measured using the Kessler 10 scales [21]. The Kessler-10 scales is validated in Ethiopia against psychiatrists’ diagnosis using the comprehensive psychopathological scale [22,23] and it contains 10 questions each containing 5-point Likert scales (1 = never, 2 = a small part of the time, 3 = some of the time, 4 = most of the times, 5 = all of the time). The K-10 instrument is a useful screening tool to identify CMD such as depression, anxiety and somatoform disorders. It asks questions regarding the presence of depression, tiredness, nervousness, restlessness, hopelessness, and worthlessness in 30 days prior to the survey. To assess the degree of mental health status of individuals, CMD was categorized as normal (score <20), moderate (score 20-24), and severe (score above 25) based on the recommendations of other authors [21,23]. Several studies have used cut off score 20 to identify cases CMD [24,25].

QoL was measured through face to face interview using the short Amharic version of the World Health Organization Quality of Life Instrument for HIV clients (WHOQOL HIV-Bref) [26]. The QoL instrument is published in the previous article [16]. In brief, it consisted of 31 Likert scale questions in six domains of QoL: Physical health (4 items); psychological well being (5 items); social relationship (4 items); environmental health (8 items); level of independence (4 items) and spiritual health (4 items). There were two general questions about general QoL and perceived general health. The overall quality of life was measured using all the questions as described in elsewhere [26].

### Data analysis

Data were analyzed using the SPSS version 16.0 and STATA® version 11 software. Items score of the K-10 were summed to create a continuous CMD outcome variable. Similarly, items scores of the WHOQOL-HIV-Bref instrument was summed to create a continuous QoL exposure variable. We used t-test and F-test to compare means of CMD between groups.

Paired-T-tests were used to compare repeated measurements of CMD at baseline and six months. Multivariate analysis was conducted using generalized estimating equations (GEE) using the Gaussian family and the identity link function. In this model, the correlations between the baseline and 6-month measurements are taken into account. Variables with significant correlation were removed. A P-value of less or equal to 0.05 was taken as the cut-off value for significance.

### Ethical considerations

Ethical clearance was obtained from the Jimma University ethical review board. Written informed consent was obtained from the study participants. To ensure confidentiality, we used codes to analyze the data. Patients who scored above 20 on the Kessler scale were referred to Psychiatry units in each hospital for further diagnosis and management.

## Results

At baseline, 465 HIV patients without TB and 124 TB/HIV co-infected patients were included. Of the co-infected patients, smear negative, smear positive and extra pulmonary TB accounted for 61 (49.2%), 42 (33.9%), 21 (16.9%) respectively. At 6 month, 540 (97 TB/HIV co infected and 455 HIV patients without TB) patients completed the follow up and 8.6% lost to follow-up. Over the six months period, 5/443(1.12%) HIV/AIDS patients developed active pulmonary TB. During the follow up period, 7.1% of the TB/HIV co-infected patient discontinued their anti-TB treatment at least once and 10 (1.8%) patients with HIV/AIDS missed at least one dose of ART (Table 1).

**Table 1:** Socio-demographic characteristics of participants at 6 months of follow up

**Variables**	**TB/HIV co-infected patients**	**HIV patients**	**P-value**
Age in years, Mean (SD)	34.5 (9.5)	33.4 (8.1)	0.227
Sex			
Male	43	185	0.505
Female	54	270	
Employment			
Unemployed	7	52	0.223
Employed	90	403	
WHO staging			
Stage I	0	71	0.000
Stage II	0	64	
Stage III	79	216	
Stage IV	18	65	
CD4 lymphocyte count, (medina)	392.6 (375.0)	383.7 (358.0)	0.762
At least missed one doses of Anti-TB or ART	7(7.1%)	10(1.8%)	-
Lost to follow-up	27	10	0.000

### Common mental disorders at baseline and 6-months

At baseline, 24.8% and 15.2% of the TB/HIV co-infected patients had severe and moderate form of CMD respectively. On the other hand, the prevalence of severe form of CMD among HIV/AIDS patients without TB at baseline and 6 month was 8.6% and 3.8% respectively. At the six month, the prevalence of severe form of CMD among TB/HIV-co-infected patients was 1.9% which showed a difference of 22.9 percentage-points. Any form of mental disorder had decreased by 36.3 percentage points in TB/HIV co-infected patients at six month compared to the baseline. Any form of CMD among HIV/AIDS patients without TB was reduced by 19.4 percentage points at the six month (Table 2).

**Table 2:** Change in common mental disorders over 6 months follow-up among participants

**Category**	**Prevalence rate among TB/HIV co-infected**		**Change from baseline to 6**^**th **^**month**	**P-value**	**Prevalence among HIV patients without TB**		**Change from baseline to 6**^**th **^**month**	**P-value**
	**At baseline (n=124)**	**6th month (n=97)**			**At baseline (n=465)**	**6th month (n=455)**		
Well	45.6	81.9	36.3	0.000	58.8	78.2	19.4	0.000
Mild disorder	14.4	9.5	−4.9		17.7	14.8	−2.9	
Moderate mental disorder	15.2	6.7	−8.5		14.9	3.1	−11.8	
Severe mental disorder	24.8	1.9	−22.9		8.6	3.8	−4.8	
Any mental disorder	54.4	18.1	−36.3		41.2	21.8	−19.4	

### Predictors of common mental disorders

QoL was strongly associated with CMD in TB/HIV co-infected patients and HIV/AIDS patients without TB (β = −0.04, P<0.001) after controlling the effect of several confounding variables such as sex, income, CD4 lymphocyte count, adherence to ART and social support (Table 3).

**Table 3:** Predictors of CMD among TB/HIV co-infected and HIV/AIDS patients without TB, Ethiopia

	**TB/HIV co-infected**		**HIV infected**	
**Variables**	**B (SE)**	**P-value**	**B (SE)**	**P-value**
**Sex**				
Male	1.00		1.00	
Female	−0.14 (0.3)	0.647	−0.03 (0.2)	0.826
**Literacy status**				
Illiterate	1.00		1.00	
Literate	−0.10 (0.4)	0.801	0.21 (0.2)	0.289
**Baseline CD4 count**				
<200	1.00		1.00	
>200	0.57 (0.3)	0.097	−0.20 (0.2)	0.198
**WHO staging**				
I	NA		1.00	
II	NA		0.21 (0.2)	0.406
III	1.00		0.04 (0.2)	0.841
VI	0.3 (0.3)	0.405	−0.08 (0.2)	0.749
**Duration on ART**	0.03 (0.03)	0.130	−0.003 (0.0)	0.587
**Adherence to ART**				
Yes	1.00		1.00	
No	−0.71 (0.6)	0.268	−0.08 (0.4)	0.840
**Adherence to anti-TB**				
Yes	1.00		NA	
No	−0.37 (0.9)	0.683		
**Social support**				
Yes	1.00		1.00	
No	−0.25 (0.4)	0.501	−0.14 (0.2)	0.481
**Source of income**				
Yes	1.00		1.00	
No	−0.11 (0.4)	0.795	0.01 (0.2)	0.967
**QoL** (WHO-QoL)	−0.04 (0.01)	0.000	−0.04 (0.0)	0.000
**Employment**				
Employed	1.00		1.00	
Unemployed	0.28 (0.6)	0.619	−0.37 (0.2)	0.118

**Figure 1:**
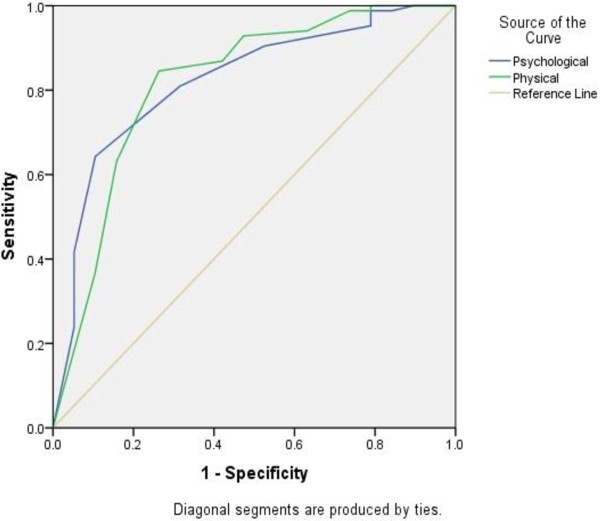
ROC curve exploring QoL change of cut off point for predicting CMD among TB/HIV co-infected and HIV patients without TB, Ethiopia (area under the curve; physical=0.818 and psychological QoL=0.821; both areas are statistically significantly from an areas of 0.5).

A ROC curve exploring the best cut-off points of physical and psychological quality of life as predictors of CMD showed that QoL is the best predictors of CMD with area under the curve of 0.82. A cut off point of 11.5 for physical and 14.0 for psychological dimension of QoL gave 80% sensitivity (Figure 1).

## Discussion

This is the first follow up study to evaluate the change in CMD among TB/HIV co-infected and HIV patients without TB who are on ART in Ethiopia. This study reveals that there is significant decline in the prevalence of CMD particularly among TB/HIV co-infected patients over a six month period. The change in CMD prevalence among TB/HIV co-infected individuals is twice than that of HIV patients without TB. Among several exposure variables, QoL is the best predictors of CMD both in co-infected patients and HIV/AIDS patients without TB.

This study reveals that the prevalence of CMD among TB/HIV co-infected and HIV patients without TB is very high. Other literatures have also documented high prevalence of mental health problems among people with HIV/AIDS [8,9] and patients with tuberculosis [12,13]. In the present study, the decline in prevalence of CMD at the 6 month of treatment and follow up was more marked among TB/HIV patients compare to people living with HIV without TB. This could be explained by two major reasons. First, in the continuation phase of anti-TB treatment (2-8 months), the physical and functional status of the patients could improve significantly which in turn brings improved mental health status of individuals. Second, perceived stigma associated with TB might also be reduced at 6 months of anti-TB treatment. Reduction in stigma might have major effect on the mental status of patients. Previous study has proven that presence of perceived stigma is highly associated with depression [16,27]. A study in Uganda has also reveals that quality of life and mental health status has improved at 12-monhts of ART [28]. In our study, continues counseling and health education given by the health care providers about adherence, diets, and self-care and prevention of opportunistic infections might contribute for the reduction of CMD in both group of patients.

In our study, QoL was the best predictor of CMD; however, CMD could also cause poorer QoL among patients with TB/HIV disease. In this study, we could not establish the temporal relationship of the two conditions. Literatures on the relationship between QoL and mental disorders are lacking worldwide. A well designed cohort or clinical trial could be helpful to establish the temporal relationship between mental disorders and QoL.

Despite significant reduction in CMD at the 6-months of follow up, CMD is still a major co-morbidity of TB/HIV disease. In a country such as Ethiopia where mental health is not an integral part of HIV/AIDS or TB care and treatment services, CMD would have negative impact on the treatment outcome of patients [6]. Mental disorders such as depression might also have negative impact on immune status of patients which counteract the benefit of ART [6].

### Limitations of the study

This study is the first of its kind to evaluate the trends in CMD among TB/HIV co-infected patients. The sample size is adequate to measure the change and the effect size. However, the follow up period is too short to analyze multiple confounders using survival analysis and Cox regression. Loss to follow up of more TB/HIV co-infected patients compared to HIV/AIDS patients without TB could also introduced bias. However, the loss to follow up group is not significantly different from the other group except in the WHO clinical stage. Most of the lost to follow-up were in WHO stage 3 and 4 (P=0.002).

## Conclusions and recommendations

The prevalence of CMD has significantly reduced particularly among TB/HIV co-infected patients over a 6 months period. However, CMD is still a major co-morbidity of TB/HIV disease. Poor QoL is the major independent predictors of CMD.

Based on the findings of this study, we recommend the integration of mental health services in TB/HIV programs. Training of health care providers at TB/HIV clinics could be helpful to screen and treat CMD among TB/HIV co-infected patients.

## Competing interests

The authors declare that they have no financial or non-financial competing interests.

## Authors’ contributions

AD conceived the study and was involved in the design, analysis and report writing. KD was involved in analysis and drafted the manuscript. AAR analyzed the data and reviewed the article. MT participated in the design and reviewed the article. YH participated in the design and critically reviewed the article. TM has reviewed the article extensively. All authors read and approved the final manuscript.

## Pre-publication history

The pre-publication history for this paper can be accessed here:

http://www.biomedcentral.com/1471-244X/13/174/prepub
